# Thermophysiological responses to occupational heat stress during simulated wildland firefighting in females and males: influence of fitness and body composition

**DOI:** 10.3389/fphys.2026.1936332

**Published:** 2026-10-27

**Authors:** Jorge Gutiérrez-Arroyo, Belén Carballo-Leyenda, Fabio García-Heras, Juan Rodríguez-Medina, Jose A. Rodríguez-Marroyo

**Affiliations:** VALFIS Research Group, Department of Physical Education and Sports, Instituto de Biomedicina (IBIOMED), Instituto de Investigación Biosanitaria de León (IBIOLEÓN), Universidad de León, León, Spain

**Keywords:** aerobic fitness, sex differences, occupational heat exposure, personal protective equipment, physiological strain, thermoregulation, core temperature, evaporative heat loss

## Abstract

This study compared thermophysiological responses to prolonged exercise during prolonged occupational heat stress in female and male participants involved in wildland firefighting activities while wearing standard personal protective equipment. Twenty-two participants (11 females and 11 males) performed a graded exercise test followed by a prolonged submaximal protocol simulating wildfire suppression tasks in a controlled environment (30 °C, 30% relative humidity). Physiological responses, gastrointestinal temperature (T_g_), chest skin temperature (T_skin_), sweat production, and heat balance components were evaluated. Females exhibited higher mean values for most physiological variables, except rating of perceived exertion, which was higher in males. However, when responses were analyzed at matched relative exercise intensities, no meaningful sex-related differences were observed in T_g_, whereas chest T_skin_ and heart rate remained higher in females. Body fat percentage was positively associated with T_g_ and chest T_skin_ (*r=*0.55 and 0.53), whereas VO_2max_ was negatively correlated with T_g_ (*r=*−0.47). This relationship disappeared when VO_2max_ was normalized to lean body mass. Heat dissipation pathways differed between groups: males showed greater evaporative heat loss, whereas females exhibited a greater contribution of dry heat exchange. These findings suggest that individual body composition and aerobic fitness may contribute to variability in thermophysiological strain during occupational uncompensable heat stress.

## 1 Introduction

Wildland firefighting is an extremely demanding occupation, primarily performed during the summer season (Rodríguez-Marroyo et al., 2012). Several factors, including complex terrain (Brotherhood, 2008), smoke exposure (Wegesser et al., 2009), the handling of heavy tools (3–20 kg) (Rodríguez-Marroyo et al., 2012), and challenging environmental conditions (Rodríguez-Marroyo et al., 2012; Carballo-Leyenda et al., 2021b) contribute to the high physical demands experienced by wildland firefighters (WFFs). Although mean ambient temperatures during wildfire suppression are usually moderate (25–34 °C) (Rodríguez-Marroyo et al., 2012; Carballo-Leyenda et al., 2021b), short-term exposure to peak temperatures approaching 120 °C and heat fluxes near ~10, 000 W·m^−2^ can occur (Carballo-Leyenda et al., 2021b). Under these circumstances, personal protective equipment (PPE) is essential for safety; however, its use substantially increases thermal strain (Carballo-Leyenda et al., 2017; Carballo-Leyenda et al., 2018) due to reduced water vapor permeability and added insulation from multilayer protective garments, which limit heat dissipation (Holmér, 2006; Carballo-Leyenda et al., 2017). This heightened thermophysiological load has been associated with performance impairments of 12–50% during firefighting-related physical tasks (Carballo-Leyenda et al., 2018; Carballo-Leyenda et al., 2021a).

Historically, physically demanding occupations have been dominated by men (Roberts et al., 2016), although female participation has increased in recent years (Anderson et al., 2022; Renberg et al., 2022). Women still represent a minority in wildland firefighting, with participation rates of approximately 10–15% in some countries (Sol et al., 2018), resulting in a limited number of studies examining their physiological responses during wildfire suppression. Given known sex-based differences in anthropometry, body composition, hormonal regulation, and sweating capacity (Cheung et al., 2000; Gagnon et al., 2009; Corbett et al., 2020), it remains important to examine whether men and women differ in their thermophysiological responses to heat stress. Understanding these differences is particularly important for developing targeted ergonomic and operational strategies that support safe and effective performance.

Traditionally, women have been considered to exhibit a less efficient thermoregulatory response due to smaller body size, higher body fat or lower aerobic fitness (Shapiro et al., 1980; Cheung et al., 2000; Havenith, 2001; Gagnon et al., 2009; Epstein et al., 2013). However, when these factors are controlled, sex differences often diminish (Gagnon et al., 2009; Charkoudian and Stachenfeld, 2014), or emerge only at high intensities, where men’s greater sweating capacity enhances heat dissipation in dry environments (Shapiro et al., 1980; Gagnon and Kenny, 2012). Conversely, women tend to exhibit lower sweat rates but greater evaporative efficiency in humid conditions (Shapiro et al., 1980). Collectively, these findings suggest that individual characteristics such as body composition and aerobic fitness may play an important role in determining thermophysiological responses during heat stress, potentially contributing to the variability observed between men and women.

Although wildland firefighting usually takes place in hot and dry environments, conditions that may favor men’s thermoregulatory responses, the humid microclimate generated beneath PPE could partially shift this balance. Relative humidity exceeding 60% has been documented under WFF clothing layers (Carballo-Leyenda et al., 2017), potentially reducing differences in thermoregulatory responses between men and women. Moreover, during uncompensable heat stress, evidence regarding sex differences becomes inconsistent (Cheung et al., 2000), with studies reporting divergent thermoregulatory responses when participants wear protective gear (McLellan, 1998; Anderson et al., 2022; Renberg et al., 2022).

While previous studies have examined the effect of protective clothing and PPE on the thermophysiological response of male WFFs (Carballo-Leyenda et al., 2017), information regarding female participants involved in wildland firefighting activities remains limited. Therefore, the purpose of this study was to describe and compare thermophysiological responses in female and male participants involved in wildland firefighting activities, and to explore the contribution of aerobic fitness and body composition to the observed responses.

## 2 Materials and methods

### 2.1 Subjects

Twenty-two Spanish participants (11 females and 11 males) involved in wildland firefighting activities were recruited through convenience sampling and voluntarily participated in the study. Participants ranged in age from 21 to 41 years (females: 21–31 years; males: 21–41 years). Because female WFFs remain underrepresented in Spain, recruitment of active female WFFs was limited. Therefore, female candidates undergoing specific training for wildland firefighting duties were included in the study. The male participants had more than 3 years of experience as active WFFs, whereas all except one of the female participants were candidates in training for WFF positions. The male participants and the active female WFF had successfully completed the Pack Test as part of their occupational selection process. The female candidates completed the same test before participating in the study to verify that they met the physical fitness requirements established for WFF personnel (Carballo-Leyenda et al., 2021a). Thus, all participants had successfully completed the Pack Test prior to the study, although their occupational experience differed. All participants engaged in regular physical activity (40–60 min per training session). Male participants typically trained 4–5 times per week, whereas female participants trained 2–3 times per week. None of the subjects reported sauna use in the months preceding the study. Female participants confirmed regular menstrual cycles and were tested during the early luteal phase (14–22 days). None were taking oral contraceptives. Written informed consent was obtained from all participants before starting the study. The study protocol was approved by the Ethics Committee (ETICA-ULE-025-2020) of the University of León (Spain) and conformed to principles of the Declaration of Helsinki.

### 2.2 Experimental design

Participants completed two laboratory-based testing sessions, separated by 48–72 h, during which they were instructed to avoid strenuous exercise (**Figure 1**). Each session consisted of a single trial, and both sessions began at approximately the same time of day (12:00–13:00 h). The first session included a graded exercise test (GXT) to determine aerobic capacity and ventilatory thresholds, as well as an assessment of body composition using multifrequency tetrapolar bioelectrical impedance (InBody 770, InBody Pvt. Ltd., Seoul, South Korea). Before the body composition assessment, participants had refrained from food and from consuming large amounts of fluid for at least 3 h and had voided prior to the measurement. In the second session, participants completed a submaximal extended test designed to assess thermophysiological responses and heat tolerance under conditions previously shown to induce progressive heat storage and limited heat dissipation in WFFs (Carballo-Leyenda et al., 2018). All tests were performed on a treadmill (h/p/cosmos pulsar, h/p/cosmos sports and medical GMBH, Nußdorf-Traunstein, Germany) while wearing the standard PPE used by Spanish WFFs (~5 kg). This PPE included thermal-resistant clothing made of 65% fire retardant viscose, 30% Nomex, and 5% Kevlar (~1.5 kg, surface density 270 g ± m^−2^, thermal resistance 0.019 m^2^·K·W^−1^, evaporative resistance 3.79 m^2^·Pa·W^−1^), as well as a helmet, neck shroud, gloves, and low-calf hiking boots. A standardized 15-min warm-up preceded each test, consisting of submaximal running at 60% of maximal heart rate (8–10 km·h^−1^) and free stretching. Participants received standardized instructions to avoid strenuous exercise, follow a carbohydrate-rich diet, and abstain from alcohol for 24 h before testing. No caffeine or tea was permitted within 2 h of testing. To promote adequate hydration before the extended test, participants were advised to consume approximately 40 mL·kg^−1^ of fluids during the preceding 24 h and an additional 500 mL of water during the 2 h before testing (Anderson et al., 2022). Pre-test hydration status was assessed via urine specific gravity (digital refractometer, PEN-WRESTLING Dip 3749, ATAGO USA Inc., Bellevue, WA, USA). Participants with values >1.020 consumed 500 mL of water 30 min before testing (Anderson et al., 2022).

**Figure 1:**
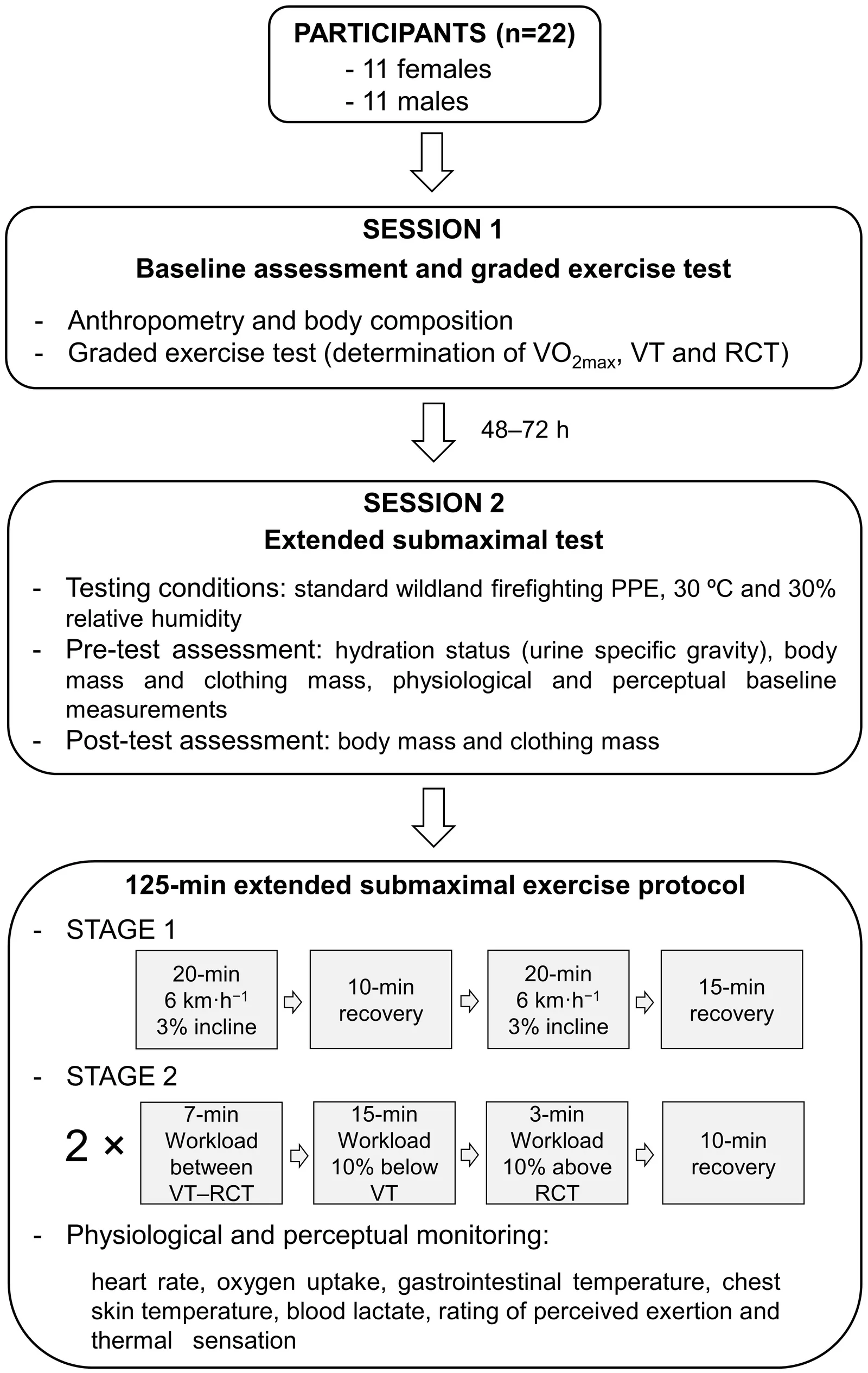
Experimental design and extended submaximal exercise protocol. VT, ventilatory threshold; RCT, respiratory compensation threshold; PPE, personal protective equipment.

### 2.3 Graded exercise test

Because the GXT was conducted while participants wore PPE, an incremental protocol was implemented to elicit maximal effort without requiring running. Testing was performed under ambient laboratory conditions (20–25 °C and 30–40% RH). The test began at 4 km·h^−1^ and 10% slope. Speed increased by 0.5 km·h^−1^ every minute until reaching 6.5 km·h^−1^, after which the slope increased by 1% per minute until volitional exhaustion. Workload (W·kg^−1^) was estimated from treadmill speed and slope as the mechanical power required for uphill locomotion and normalized to body mass. This protocol resulted in an average workload increment of ~0.16 W·kg^−1^ per minute. Heart rate (HR) and respiratory gas exchange were continuously recorded using a 12-lead ECG and a breath-by-breath analyzer (Medisoft Ergocard Professional, Medisoft Group, Sorinnes, Belgium), calibrated before each test following the guidelines provided by the manufacturer. Rating of perceived exertion (RPE) was obtained at the end of each stage using the Borg 6–20 scale (Borg, 1982). Maximal workload was defined as the highest workload attained during the test and was calculated as the workload corresponding to the last fully completed stage plus the proportional workload completed during the final uncompleted stage. VO_2max_ and maximal HR were defined as the highest 30-s averages before exhaustion. VO_2max_ was considered valid when at least two criteria were met (Midgley et al., 2009): VO_2_ plateau (≤150 mL·min^−1^), RER ≥1.15, maximal HR within ±10 beats of age-predicted maximal HR (220-age), and/or RPE ≥18. Ventilatory (VT) and respiratory compensation thresholds (RCT) were identified according to previously established criteria (Davis, 1985). Two independent researchers determined the thresholds, and disagreements were resolved by a third evaluator.

### 2.4 Submaximal extended test

The protocol was designed to simulate the sustained metabolic and thermal demands associated with wildfire suppression while maintaining controlled laboratory conditions (Rodríguez-Marroyo et al., 2012). The test included two exercise stages separated by 15 min of passive recovery. In the first stage, participants completed two 20-min bouts of walking at 6 km·h^−1^ and 3% incline, separated by 10 min of passive recovery. This workload represented the intensity at which WFFs typically hike (~0.5 W·kg^−1^) (Sol et al., 2018) and simulates the demands of suppression tasks (Carballo-Leyenda et al., 2017). During the second exercise stage, participants performed two 25-min bouts with varying intensities, separated by 10 min of passive recovery. Each bout included: 7 min between VT and RCT (workload at VT + 50% of the VT-RCT difference), 15 min at a workload 10% below VT, and 3 min at a workload 10% above RCT (**Figure 2**). This distribution replicated intensity patterns during 1–3 h wildfire suppressions (~30, ~60 and ~10%, respectively) (Rodríguez-Marroyo et al., 2012). Total test duration was 125 min, excluding instrumentation and preparation procedures, with a work-to-rest ratio similar to real deployments (Rodríguez-Marroyo et al., 2012). Ambient temperature and relative humidity were monitored throughout testing using a Kestrel 5400FW Fire Weather Meter Pro (Nielsen-Kellerman, Boothwyn, PA, USA; accuracy: ± 0.5 °C and ±3% RH, respectively). Environmental conditions during exercise were maintained at 30 °C and 30% RH, corresponding to environmental conditions frequently encountered during wildfire suppression operations in Mediterranean environments (Rodríguez-Marroyo et al., 2012). During recovery periods, ambient conditions were maintained at 23 °C and 44% RH. Subjects were allowed to drink water ad-libitum at 15 °C during these periods.

**Figure 2:**
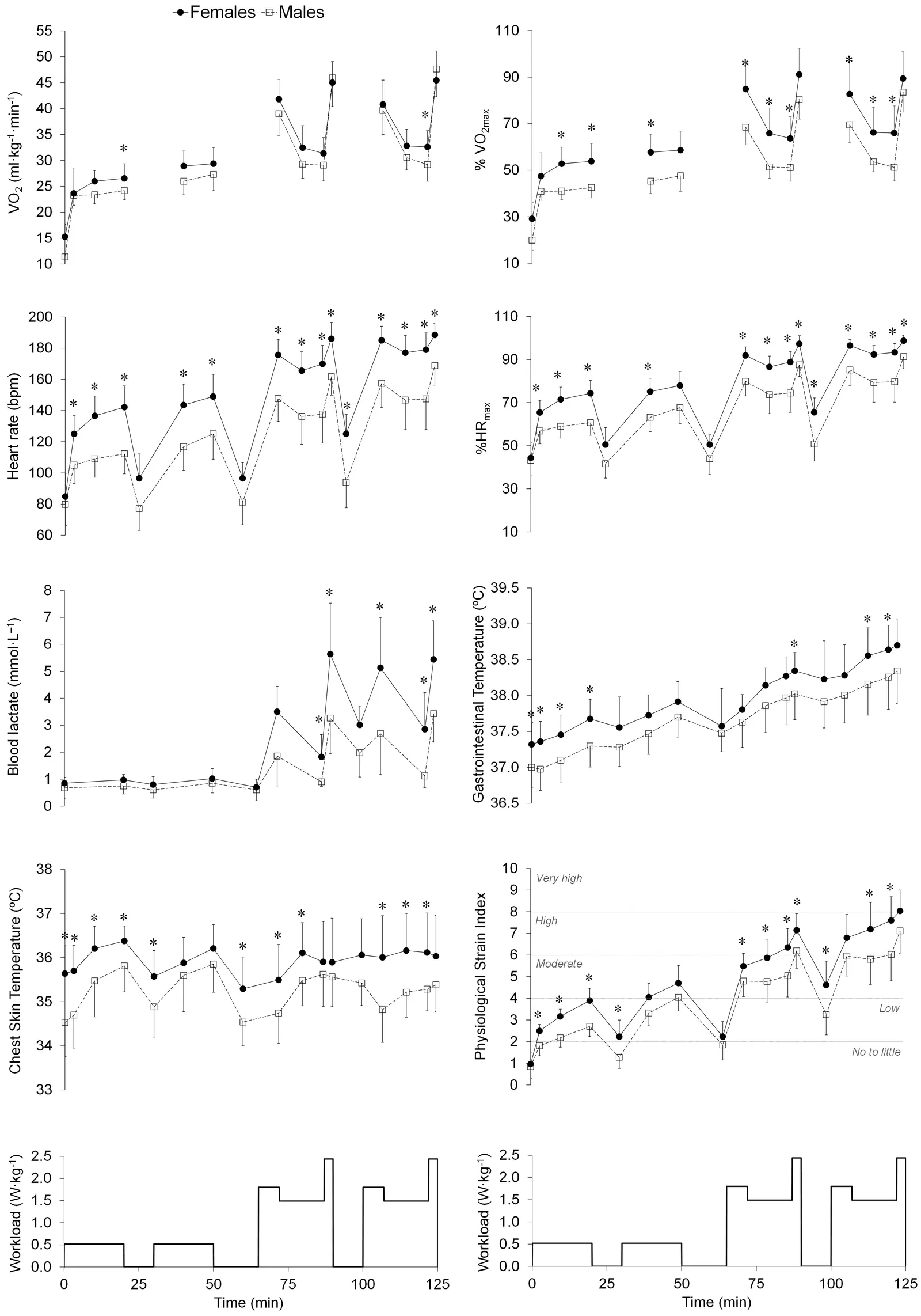
Comparative response of the thermophysiological variables during the submaximal extended test. Values are mean ± SD. *, significant difference between sexes (*p* < 0.05).

The HR was recorded every 5 s (RS800, Polar Electro Oy, Kempele, Finland). Respiratory variables were recorded breath-by-breath only during exercise bouts. Gastrointestinal temperature (T_g_) was measured every 30 s using an ingestible telemetry pill (e-Celsius Performance, Bodycap, Hérouville Saint-Clair, France), ingested ≥8 h before testing (Larsen et al., 2015). Chest T_skin_ was measured every minute using an iButton sensor (DS1922L-F50, Maxim Integrated, Sunnyvale, California, USA) attached to the left major pectoral muscle (Fixomull, BSN Medical, Hamburg, Germany). HR and T_g_ data were averaged every minute to compute the physiological strain index (PSI) (Tikuisis et al., 2002). Data from the last 3 min of each workload segment were considered for the analysis. Blood lactate concentration was assessed after each exercise intensity bout using earlobe capillary samples (Lactate Scout, Senslab, Leipzig, Germany). RPE was obtained using the Borg 6–20 scale (Borg, 1982) by the same male researcher. Thermal sensation (TS) was rated using the 9-point scale proposed by Havenith and Heus (2004), ranging from 1 to 9, with the verbal anchors “comfortably warm”, “uncomfortably warm”, “hot”, and “very hot” corresponding to values of 2, 4, 6, and 8, respectively. Intermediate values represented sensations between the corresponding verbal anchors.

Subjects’ body mass was measured in underwear, and all clothing components were individually weighed (50K150, COBOS, Hospitalet de Llobregat, Barcelona, Spain) before testing and immediately after completion of the 125-min protocol. Participants remained fully dressed in the PPE throughout the protocol, including the recovery periods. Changes in body and clothing mass were used to estimate total sweat production, sweat retained in the clothing, and sweat evaporation (Kofler et al., 2015; Larsen et al., 2015), following procedures previously used in wildland firefighters (Carballo-Leyenda et al., 2017; Carballo-Leyenda et al., 2018), with total sweat production corrected for fluid intake. Sweat evaporative efficiency was calculated as the ratio of evaporated sweat to total sweat produced (Kofler et al., 2015; Larsen et al., 2015).

Finally, heat balance components (W·m^−2^) were estimated using the partitional calorimetry model proposed by Bröde et al. (2008). Briefly, metabolic heat production, heat storage, evaporative heat loss, respiratory heat exchange and dry heat exchange were calculated from physiological, anthropometric and environmental measurements. Net metabolic heat production was determined from VO_2_, respiratory quotient, body surface area (DuBois formula) and mechanical work (McLellan et al., 1996). Heat storage was estimated from the rate of change in body temperature. Mean body temperature was estimated using T_g_ and chest T_skin_, with chest T_skin_ used as a surrogate for mean skin temperature, following the approach described by Kofler et al. (2015). Evaporative heat loss was calculated from sweat evaporation, whereas respiratory heat exchange was estimated from environmental conditions and metabolic heat production. Dry heat exchange was estimated as the residual term of the heat balance equation (Bröde et al., 2008).

### 2.5 Statistical analyses

Data are presented as mean ± standard deviation (*SD*). Normality was assessed using the Shapiro-Wilk test. Non-normal variables were log-transformed prior to analysis. An independent *t*-test was employed to compare anthropometric and physiological variables between sexes, as well as sweat measurements and heat balance components. Rates of change were calculated using within-individual linear regression excluding recovery periods. The resulting regression equations were used to estimate T_g_, chest T_skin_, HR, and PSI values at standardized relative exercise intensities ranging from 40 to 80% of VO_2max_. Only interpolation within the range of exercise intensities attained by each participant was performed. The upper limit was defined by the lowest maximum relative exercise intensity attained among participants to avoid extrapolation beyond the observed data. Sex differences at matched exercise intensities were analyzed, using two-way repeated-measures ANOVA (sex × exercise intensity). Additionally, an ANOVA (sex × time) assessed differences during the extended test. Bonferroni’s test was applied for *post-hoc* comparisons when a significant *F* value was obtained. In addition, Pearson correlation analyses were performed to explore the potential contribution of body composition and aerobic fitness variables to thermophysiological responses during the extended test. Cohen’s *d* and partial eta-squared (η_p_^2^) were calculated as the effect size measures for *t*-tests and ANOVA, respectively. Statistical analysis was performed using SPSS+ V.26.0 software (Chicago, Illinois, USA).

## 3 Results

**Table 1** shows the anthropometric and physiological characteristics of the participants. Males exhibited a larger body surface area, although this difference was attenuated when normalized to body mass. Females had a higher percentage of body fat, whereas males exhibited a higher VO_2max_ and achieved greater maximal workload, as well as higher workloads at both RCT and VT.

**Table 1:** Anthropometric and physiological characteristics of the subjects (mean ± *SD*).

Variable	Females	Males	Cohen’s *d* (95% CI)
Age (years)	23.9 ± 3.2	31.9 ± 6.6*	−1.54 (−2.43, −0.54)
Height (cm)	163.8 ± 3.4	178.8 ± 5.8*	−3.15 (−4.26, −1.81)
Weight (kg)	62.7 ± 9.1	73.9 ± 7.7*	−1.33 (−2.20, −0.36)
BMI (kg·m^−2^)	23.3 ± 3.3	23.0 ± 1.7	0.11 (−0.73, 0.95)
BSA (m^2^)	1.7 ± 0.1	1.9 ± 0.1*	−2.00 (−2.93, −0.91)
BSA·kg^-1^ (cm^2^·kg^−1^)	2.7 ± 0.2	2.6 ± 0.1	0.63 (−0.25, 1.46)
Body fat (%)	24.8 ± 5.0	12.5 ± 3.2*	2.93 (1.64, 4.00)
VO_2max_ (mL·kg^−1^·min^−1^)	47.0 ± 8.0	55.0 ± 7.3*	−1.04 (−1.89, −0.12)
VO_2max_ (mL·kg^−1^ LBM·min^−1^)	62.4 ± 9.3	62.9 ± 8.0	−0.06 (−0.89, 0.78)
Maximal workload (W·kg^−1^)	2.6 ± 0.3	3.5 ± 0.4*	−2.55 (−3.55, −1.34)
Maximal workload (W·kg^−1^ LBM)	3.3 ± 0.3	4.0 ± 0.5*	−1.70 (−2.60, −0.67)
Workload at RCT (W·kg^−1^)	2.1 ± 0.2	2.7 ± 0.4*	−1.90 (−2.82, −0.83)
Workload at RCT (W·kg^−1^ LBM)	2.6 ± 0.3	3.0 ± 0.5*	−0.97 (−1.81, −0.05)
Workload at VT (W·kg^−1^)	1.6 ± 0.1	1.9 ± 0.3*	−1.34 (−2.21, −0.37)
Workload at VT (W·kg^−1^ LBM)	2.0 ± 0.2	2.1 ± 0.4	−0.32 (−1.14, 0.54)

BMI, body mass index; BSA, body surface area; VO_2max_, maximal oxygen uptake. RCT, respiratory compensation threshold; VT, ventilatory threshold; LBM, lean body mass; 95% CI, 95% confidence interval. *, significant difference between sexes (*p* < 0.05).

When examining the thermophysiological response during the extended submaximal test, main effects of time were observed for all monitored variables (F (16, 320) = 12.97–463.76, η_p_^2^ = 0.41–0.96) (**Figures 2** and **3**). A main effect of sex was also found for all variables (F (1, 20) = 5.03–16.45, η_p_^2^ = 0.23–0.46), except for VO_2_ and TS. Females exhibited higher mean values for all monitored variables except RPE, which was higher in males. The rate of increase in RPE across the test was also higher in males (**Table 2**). Conversely, VO_2_, HR and blood lactate increased more rapidly in females. Although T_g_, chest T_skin_ and PSI differed between groups, the rate of increase for these variables was similar (**Table 2**). Notably, these variables, together with TS, were the only outcomes not influenced by a sex × time interaction.

**Figure 3:**
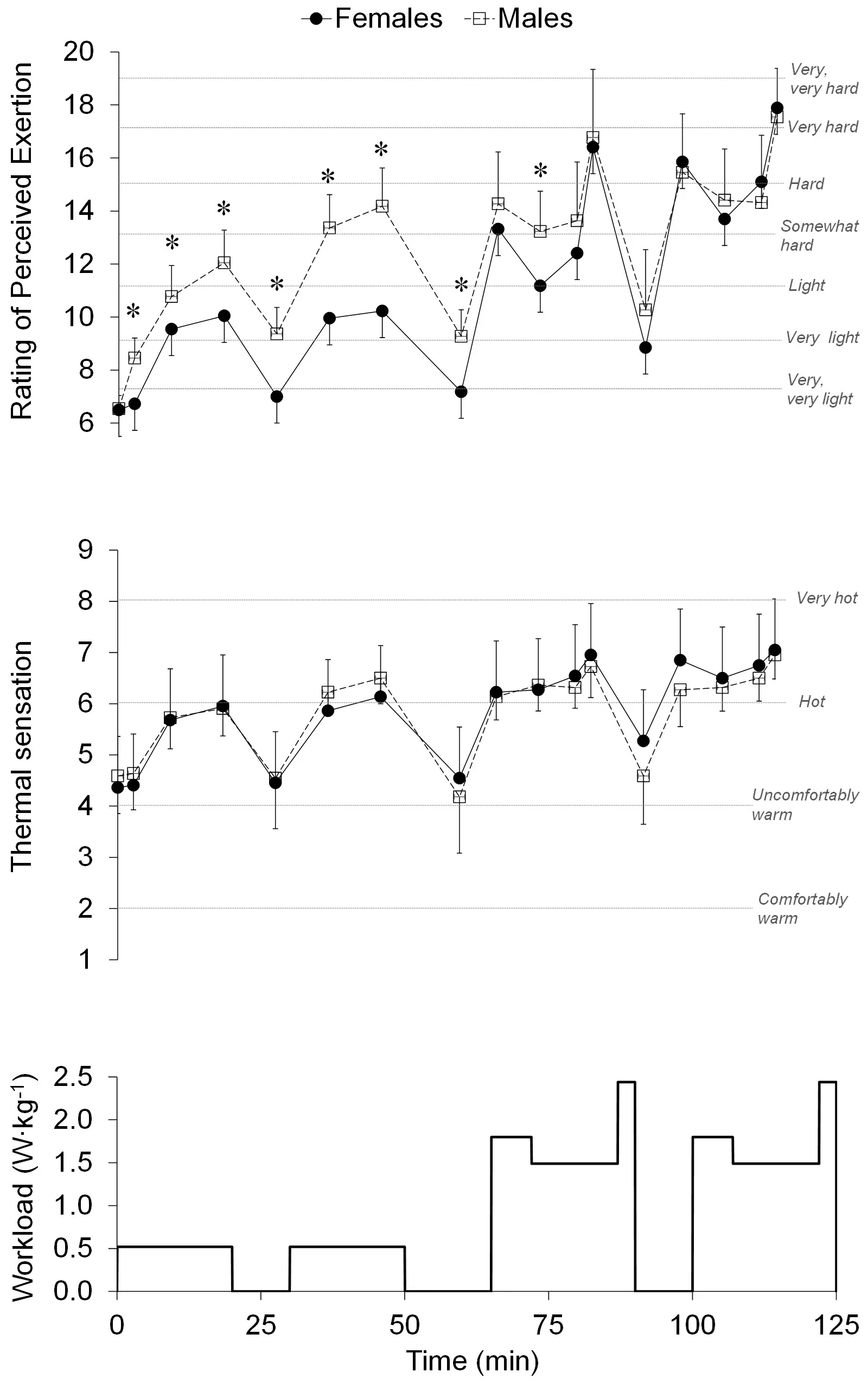
Rating of perceived exertion and thermal sensation throughout the submaximal extended test. Values are mean ± SD. *, significant difference between sexes (*p* < 0.05).

**Table 2:** Mean values and rate of change per minute (ROC) for physiological variables in the submaximal extended test.

Variable	Females		Males		η_p_^2^
	mean ± *SD*	ROC	mean ± *SD*	ROC	
VO_2_ (ml·kg^−1^·min^−1^)	31.7 ± 3.2	0.16 ± 0.03	30.4 ± 2.8	0.13 ± 0.02*	0.00
VO_2_ (ml·kg^−1^ LBM·min^−1^)	40.1 ± 4.4	0.20 ± 0.05	34.8 ± 3.0*	0.15 ± 0.03*	0.26
Percentage of VO_2max_ (%)	64.9 ± 7.4	0.33 ± 0.09	54.6 ± 5.1*	0.23 ± 0.04*	0.27
Heart rate (ppm)	152 ± 10	0.61 ± 0.07	128 ± 14*	0.50 ± 0.10*	0.46
Percentage of HR_max_ (%)	79.7 ± 3.5	0.32 ± 0.05	67.0 ± 6.5*	0.27 ± 0.05*	0.43
Blood lactate (mmol·L^−1^)	3.1 ± 0.7	0.52 ± 0.20	2.1 ± 0.7*	0.16 ± 0.08*	0.46
T_g_ (°C)	38.0 ± 0.2	0.01 ± 0.00	37.7 ± 0.3*	0.01 ± 0.00	0.26
Chest Skin Temperature (°C)	35.9 ± 0.6	0.001 ± 0.008	35.3 ± 0.6*	0.001 ± 0.005	0.28
PSI	5.1 ± 0.6	0.04 ± 0.01	4.2 ± 0.7*	0.03 ± 0.01	0.24
Thermal sensation	6.0 ± 0.5	0.02 ± 0.01	5.9 ± 0.3	0.01 ± 0.01	0.00
RPE	11.4 ± 1.2	0.07 ± 0.02	13.0 ± 1.0*	0.06 ± 0.01*	0.28

VO_2_, oxygen uptake; LBM, lean body mass; HR_max_, maximal heart rate; T_g_, gastrointestinal temperature; PSI, physiological strain index; RPE, ratings of perceived exertion. Values are mean ± *SD*. ηp^2^ corresponds to the main effect of group (female *vs.* male). *, significant difference between sexes (*p* < 0.05).

Maximal T_g_ reached during the test was higher in females than males (38.8 ± 0.3 *vs.* 38.4 ± 0.4 °C). However, maximal PSI values were comparable between females (7.9 ± 1.2) and males (7.4 ± 1.3), and no significant sex differences were observed in maximal chest T_skin_ (36.7 ± 0.4 *vs.* 36.4 ± 0.6 °C). When temperature data were adjusted for the same relative exercise intensity (**Figure 4**), between-group differences were small for T_g_ (F (1, 20) = 0.40, *p* = 0.54, η_p_^2^ = 0.02) and absent for PSI (F (1, 20) = 0.00, *p* = 0.97, η_p_^2^ = 0.00). In contrast, between-group differences remained for chest T_skin_ (F (1, 20) = 6.72, *p* = 0.02, η_p_^2^ = 0.26) and HR (F (1, 20) = 4.48, *p* = 0.04, η_p_^2^ = 0.18) after adjustment for relative exercise intensity.

**Figure 4:**
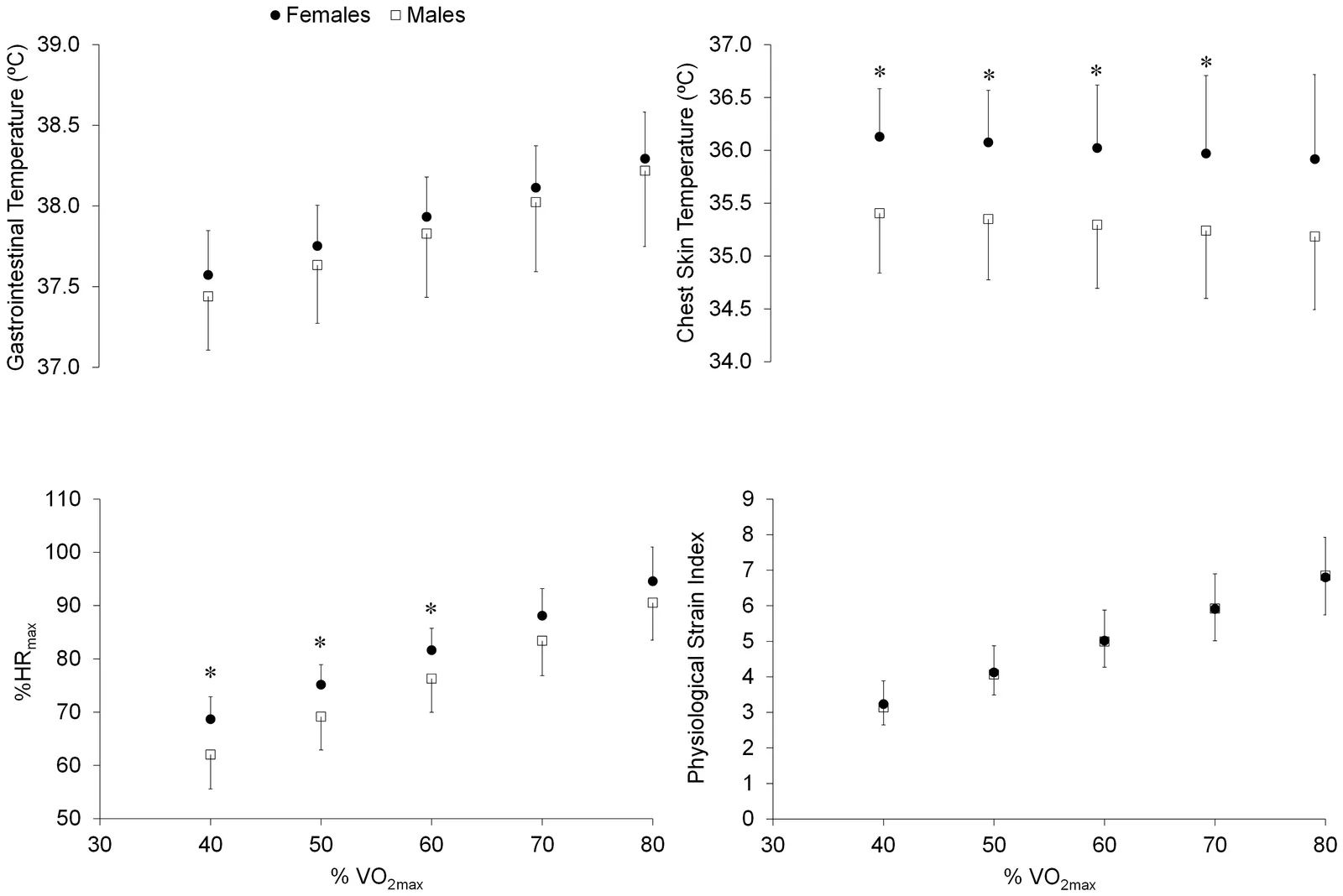
Interpolated values of gastrointestinal temperature, chest temperature, heart rate and physiological strain index for exercise intensities ranging from 40 to 80 % of VO_2max_. Values are mean ± SD. *, significant difference between sexes (*p* < 0.05).

Exploratory correlation analyses showed associations between VO_2max_ (ml·kg^−1^·min^−1^) and body fat percentage (*r = −*0.43), exercise intensity during the extended test (*r = −*0.47 and *−*0.50 for %VO_2max_ and %HR_max_, respectively), mean (*r =* −0.47) and maximal T_g_ (*r =* −0.53), PSI (*r =* −0.58), and the rate of increase in VO_2_ (*r =* −0.45) and blood lactate (*r =* −0.43). When VO_2max_ was normalized to lean body mass, no associations were observed. Body fat percentage showed associations with exercise intensity (*r =* 0.71 and 0.62 for %VO_2max_ and %HR_max_, respectively), mean (*r =* 0.55) and baseline T_g_ (*r =* 0.56), mean (*r =* 0.53) and baseline chest T_skin_ (*r =* 0.41), and PSI (*r =* 0.61). Body fat was also associated with the rates of increase in exercise intensity (*r =* 0.79 and 0.46 for %VO_2max_ and %HR_max_, respectively), blood lactate (*r =* 0.69), and PSI (*r =* 0.49) during the extended test.

Total sweat production (1.4 ± 0.3 *vs.* 2.3 ± 0.4 kg, *d* = −2.55 [−3.55, −1.34]) and sweat evaporation (1.1 ± 0.2 *vs.* 1.5 ± 0.4 kg, *d* = −1.26 [−2.13, −0.31]) were higher in male than female WFFs. However, when normalized to body surface area (670.2 ± 134.6 *vs.* 773.3 ± 176.9 g·m^−2^, *d* = −0.66 [−1.49, 0.23]) or body mass (18.1 ± 4.1 *vs.* 20.2 ± 4.6 g·kg^−1^, *d* = −0.48 [−1.31, 0.38]), differences in sweat evaporation were attenuated. Females exhibited greater sweat evaporative efficiency (82.2 ± 10.3 *vs.* 63.1 ± 10.6%, *d* = 1.83 [0.77, 2.74]). Regarding heat balance components, heat production and heat storage were similar between groups, but heat loss pathways differed (**Table 3**). Evaporative heat loss from the skin was greater in males, whereas dry heat exchange was higher in females.

**Table 3:** Estimated parameters of heat balance (mean ± *SD*).

Variable	Females	Males	Cohen’s *d* (95% CI)
Heat storage (W·m^−2^)	20.3 ± 9.2	22.7 ± 5.2	−0.32 (−1.15, 0.53)
Metabolic heat production (W·m^−2^)	360.7 ± 38.4	363.5 ± 25.9	−0.09 (−0.92, 0.75)
Respiratory heat exchange (W·m^−2^)	32.2 ± 3.3	32.7 ± 2.1	−0.18 (−1.01, 0.66)
Evaporative heat loss (W·m^−2^)	217.3 ± 43.7	263.6 ± 46.1*	−1.03 (−1.88, −0.11)
Dry heat exchange (W·m^−2^)	92.5 ± 49.4	48.5 ± 21.4*	1.16 (0.21, 2.01)

95% CI, 95% confidence interval. *, significant difference between sexes (*p* < 0.05).

## 4 Discussion

This study is the first to compare thermophysiological responses to prolonged occupational heat stress in male and female individuals involved in wildland firefighting activities while wearing standard PPE. The findings indicate that although females exhibited a more pronounced response in most physiological variables, their overall thermoregulatory capacity remained effective, as no meaningful sex differences were observed in T_g_ or heat storage. Moreover, when responses were analyzed at matched relative exercise intensities, no sex differences were observed in T_g_, suggesting that core thermal responses were largely influenced by relative workload. Nevertheless, the pathways through which heat was dissipated differed between sexes, with females showing a greater contribution of dry heat loss and males relying more heavily on evaporative heat loss.

The higher physiological strain observed in females might be partly explained by their anthropometric and physiological characteristics. Our results show that females exhibited lower aerobic capacity than males (**Table 1**), consistent with previous findings reporting lower VO_2max_ values in women compared with men in both civilian and military populations (McLellan, 1998; Gagnon et al., 2009; Corbett et al., 2020). The lower aerobic capacity of the female group may have contributed to the higher relative exercise intensity observed during the prolonged test, which was reflected in higher mean HR and a greater percentage of VO_2max_ during the submaximal exercise stages. Lower aerobic fitness has previously been associated with greater physiological strain and an increased risk of heat-related fatigue and illness during prolonged work in hot environments (Epstein et al., 2013; Corbett et al., 2020).

Interestingly, despite experiencing higher cardiovascular and metabolic strain, females reported lower perceived exertion than males (**Table 2**). Previous studies examining sex-related differences in RPE have reported inconsistent findings, ranging from lower RPE in females at comparable relative exercise intensities (Cook et al., 1998) to no sex differences when exercise intensity was expressed relative to individual capacity (Garcin et al., 2005). Previous athletic experience may also influence RPE responses and their correspondence with physiological indicators (Winborn et al., 1988), which could be relevant given the differences in firefighting experience between groups in the present study. In addition, differences between the sex of participants and observers have previously been proposed as a potential factor influencing RPE responses (Rodríguez-Marroyo et al., 2014), while experimental evidence indicates that the sex of the experimenter can influence reported RPE under some exercise conditions (Boutcher et al., 1988). Thus, as RPE was collected by a male researcher in the present study, the evaluator-participant context may have potentially influenced the reported RPE. However, these potential explanations were not directly assessed in the present study and should be confirmed in future studies specifically designed to examine their influence on RPE.Sex differences in cardiovascular strain and chest T_skin_ were more evident when responses were analyzed at matched exercise intensities (**Figure 4**). Females exhibited higher HR and chest T_skin_ up to ~70% of VO_2max_ for similar T_g_ and PSI values. This pattern is consistent with greater redistribution of body heat towards the skin in females, potentially contributing to the maintenance of comparable core thermal responses despite greater cardiovascular strain. However, skin blood flow and regional heat transfer were not directly measured in the present study. Such a thermophysiological profile may be related to the lower sweating rates typically reported in women, which may reduce evaporative heat loss and increase reliance on dry heat transfer mechanisms such as convection and conduction (Gagnon and Kenny, 2012; Notley et al., 2019; Anderson et al., 2022).

In agreement with this interpretation, females demonstrated lower total sweat production than males but significantly greater evaporative efficiency (**Table 3**). These results are consistent with previous studies indicating that women typically produce less sweat per gland but may display greater evaporative efficiency under certain conditions (Havenith, 2002; Gagnon et al., 2008). Although garment fit was not directly assessed in the present study, differences in PPE fit might have further amplified this effect. Standard protective clothing is typically designed based on male anthropometric characteristics (McQuerry, 2020), which may lead to a looser fit in female WFFs. Such looseness can create air pockets between the skin and the garment, potentially enhancing ventilation and facilitating sweat evaporation (Boorady et al., 2013). However, poorly fitted PPE may also impair mobility (Coca et al., 2010; Boorady et al., 2013) and increase burn injury risk under real fire conditions due to the bellows effect (Keiser and Rossi, 2008). These findings highlight the importance of developing PPE adapted to female anthropometrics in order to improve both thermal tolerance and operational safety.

Despite these differences in heat dissipation pathways, no relevant sex differences were observed in core thermal responses during the prolonged test. T_g_ and PSI increased similarly over time in both sexes (**Table 2**; **Figure 3**), suggesting that the overall thermal strain imposed by the protocol was comparable. Nevertheless, approximately half of the participants (7 females and 3 males) exceeded a T_g_ of 38.5 °C during the test, indicating that the protocol induced substantial physiological strain. Similar T_g_ responses have been reported in previous studies involving firefighters performing prolonged work in hot environments (Anderson et al., 2022). Previous studies have suggested that thermophysiological responses may diverge at higher heat loads and exercise intensities (Gagnon and Kenny, 2012). In the present study, the observed responses appeared to be influenced not only by sex, but also by individual anthropometric and physiological characteristics.

One female participant in this study terminated the test after reaching a T_g_ of 39 °C and experiencing dizziness. This participant presented a relatively high body mass (78.3 kg), body mass index (27.8 kg·m^−2^) and body fat percentage (28.3%), as well as lower aerobic capacity (42 ml·kg^−1^·min^−1^) compared with the rest of the sample. Higher adiposity, elevated body mass index and lower aerobic fitness have previously been associated with reduced heat tolerance due to impaired heat dissipation and greater metabolic strain during exercise (Cheung et al., 2000; Havenith, 2001; Alele et al., 2021). This individual case is consistent with a possible contribution of morphological and fitness-related characteristics to heat tolerance, although no causal inference can be drawn from a single observation.

Consistent with this observation, the exploratory correlation analyses suggest that individual physiological and morphological characteristics may contribute to thermophysiological responses during occupational heat stress. Higher aerobic fitness (VO_2max_) was associated with lower relative exercise intensity and lower thermophysiological strain, as reflected by negative correlations with mean and maximal T_g_, PSI, and the rates of increase in metabolic variables during the test. In contrast, body fat percentage showed positive associations with exercise intensity, T_g_, chest T_skin_, and PSI. These associations are consistent with previous research indicating that higher body fat may impair heat dissipation due to the lower thermal conductivity of adipose tissue (McLellan, 1998; Havenith, 2001), while greater aerobic fitness may improve cardiovascular stability and tolerance to prolonged exercise in hot environments (Shapiro et al., 1980; Cheung et al., 2000; Alele et al., 2021). Interestingly, the associations involving VO_2max_ were not observed when aerobic capacity was normalized to lean body mass, suggesting a possible contribution of body composition to the observed relationships. However, given the exploratory nature of these analyses, the small sample size, and the absence of adjustment for multiple comparisons, these findings should be interpreted cautiously and require confirmation in larger studies. Overall, the observed associations suggest that aerobic fitness and body composition may contribute to individual variability in thermophysiological responses, although their independent contributions cannot be established from the present data.

Although baseline T_g_ was slightly higher in females than in males, this difference may be partly explained by hormonal fluctuations associated with the menstrual cycle. Core temperature can be elevated by up to ~0.8 °C during the luteal compared with the follicular phase, primarily due to the thermogenic effect of progesterone, which also shifts the onset thresholds for sweating and cutaneous vasodilation toward higher core temperatures (Charkoudian and Stachenfeld, 2016; Baker et al., 2020). Importantly, evidence suggests that this higher initial core temperature may persist during exercise without necessarily being accompanied by differences in sweat rate or skin temperature (Baker et al., 2020). In the present study, all female participants were tested during the early luteal phase, which may therefore have contributed to their higher baseline T_g_. Testing during the early follicular phase could have minimized this progesterone-related elevation and facilitated comparison with males. However, menstrual cycle phase was determined by self-report rather than hormonal verification, and therefore the actual hormonal status of the participants cannot be confirmed. Future studies should verify menstrual cycle phase using hormonal measurements and consider testing during the early follicular phase when the objective is to minimize the influence of reproductive hormones on baseline core temperature. During the prolonged test, females also showed slightly higher T_g_ values. This difference may reflect both their higher baseline T_g_ and the greater physiological strain associated with performing the protocol at a higher relative exercise intensity. At matched relative exercise intensities (**Figure 4**), no clear between-group differences in T_g_ were observed. These findings suggest that relative exercise intensity may have contributed to the differences observed during the prolonged test, although the potential influence of menstrual cycle phase cannot be excluded.

No sex differences were observed in thermal sensation during the protocol. Similar findings have been reported by Renberg et al. (2022), who observed comparable perceptual and physiological responses between men and women during a heat tolerance test performed while wearing firefighting protective clothing. These results suggest that, when heat loss is similarly constrained by protective equipment, perceptual responses to heat stress may be comparable between sexes. This highlights the importance of considering both physiological and perceptual responses when designing strategies for heat management in wildfire suppression activities.

This study presents several limitations. First, while the male participants were experienced WFFs, most female participants were candidates undergoing training, which may have influenced aerobic fitness and familiarity with PPE. Therefore, the observed between-group differences cannot be attributed exclusively to biological sex. Nevertheless, the characteristics of the female group may reflect those of women preparing to enter operational wildland firefighting service, providing applied relevance to this population while limiting the generalization of the findings to experienced female WFFs. Second, although menstrual cycle phase was considered, hormonal status was based on self-report rather than direct hormonal measurements. Third, the relatively small sample size limits statistical power, particularly for detecting sex × time and sex × exercise intensity interactions, as well as the generalizability of the findings. In addition, the correlation analyses were exploratory in nature, and no adjustment for multiple comparisons was applied. Given the number of associations examined, the possibility of Type I error cannot be excluded. Therefore, these associations should be interpreted cautiously and require confirmation in larger studies. Fourth, PPE fit was not quantitatively assessed, despite its potential influence on heat exchange and movement efficiency. Furthremore, chest T_skin_ was measured at a single site and used as a surrogate for mean T_skin_ in the heat balance calculations. This approach may not adequately capture regional differences in T_skin_, particularly under PPE, where local microclimates may develop. Consequently, the estimated heat balance components, including heat storage, should be interpreted with caution. Future studies should incorporate multi-site skin temperature measurements to improve the accuracy of these estimates. In addition, sweat production and evaporation were estimated from changes in body and clothing mass. Although participants remained fully dressed throughout the protocol and clothing was weighed immediately after exercise, sweat lost through dripping was not collected and may have introduced some error into these estimates. Finally, the findings should be interpreted within the context of the age range and characteristics of the participants studied, as well as the controlled laboratory conditions employed. Therefore, caution is warranted when extrapolating the present results to different firefighter populations, environmental conditions, wildfire scenarios, or seasonal contexts.

## 5 Conclusion

Although females experienced greater cardiovascular strain and higher chest T_skin_, males produced more sweat whereas females exhibited greater evaporative efficiency. Despite these differences, no clear between-group differences in T_g_ or heat storage were observed, although heat dissipation pathways differed, with males showing greater evaporative heat loss and females a greater contribution of dry heat exchange. Exploratory associations suggest that aerobic fitness and body composition may contribute to individual variability in thermophysiological responses during occupational heat stress. However, their independent contributions and their relative importance compared with biological sex cannot be established from the present study. These findings support further investigation of individual fitness, body composition, and PPE characteristics when evaluating heat tolerance in individuals involved in wildland firefighting activities. Future studies involving larger and more comparable groups should clarify these relationships and investigate strategies to optimize PPE fit, training, and occupational heat management.

## Data Availability

The data presented in this study are available on request from the corresponding author. The data are not publicly available due to privacy restrictions.
